# Supplementary material for: Divergent neural circuits for proprioceptive and exteroceptive sensing of the Drosophila leg
Source: Nat Commun. 2025 May 2;16:4105. doi: 10.1038/s41467-025-59302-3 (PMC12048489; doi:10.1038/s41467-025-59302-3)
Supplement: Supplementary file 2 — Reporting Summary [file 41467_2025_59302_MOESM2_ESM.pdf]

nature portfolio

Corresponding author(s): Sweta Agrawal

Last updated by author(s): Feb 6, 2025

Reporting Summary

Nature Portfolio wishes to improve the reproducibility of the work that we publish. This form provides structure for consistency and transparency in reporting. For further information on Nature Portfolio policies, see our Editorial Policies and the Editorial Policy Checklist.

Statistics

For all statistical analyses, confirm that the following items are present in the figure legend, table legend, main text, or Methods section.

n/a	Confirmed	
☐	☒	The exact sample size (*n*) for each experimental group/condition, given as a discrete number and unit of measurement
☒	☐	A statement on whether measurements were taken from distinct samples or whether the same sample was measured repeatedly
☒	☐	The statistical test(s) used AND whether they are one- or two-sided<br>*Only common tests should be described solely by name; describe more complex techniques in the Methods section.*
☒	☐	A description of all covariates tested
☒	☐	A description of any assumptions or corrections, such as tests of normality and adjustment for multiple comparisons
☐	☒	A full description of the statistical parameters including central tendency (e.g. means) or other basic estimates (e.g. regression coefficient) AND variation (e.g. standard deviation) or associated estimates of uncertainty (e.g. confidence intervals)
☒	☐	For null hypothesis testing, the test statistic (e.g. *F*, *t*, *r*) with confidence intervals, effect sizes, degrees of freedom and *P* value noted<br>*Give P values as exact values whenever suitable.*
☒	☐	For Bayesian analysis, information on the choice of priors and Markov chain Monte Carlo settings
☒	☐	For hierarchical and complex designs, identification of the appropriate level for tests and full reporting of outcomes
☒	☐	Estimates of effect sizes (e.g. Cohen's *d*, Pearson's *r*), indicating how they were calculated

Our web collection on statistics for biologists contains articles on many of the points above.

Software and code

Policy information about availability of computer code

Data collection	Data presented in the paper was analyzed from the CAVE materialization v604 timestamp 1684915801.222989. Annotated connectivity matrices (Figure 2) will be available as Pandas data frames (https://pandas.pydata.org/) at the GitHub repository: https://github.com/sagrawal/Lee_2024. Links to public segmentations are available throughout the text, as well as in a document at the git-hub repository.
Data analysis	All data analysis scripts are available in the GitHub repository associated with the manuscript, https://github.com/sagrawal/Lee_2024. This repository includes scripts to recreate the analyses and figures in the paper, as well as scripts to recreate the connectivity matrices for users authorized to interact with the CAVEclient. All analysis was performed in Python 3.9 using custom code, making extensive use of CAVEclient (https://github.com/seung-lab/CAVEclient) and CloudVolume to interact with data infrastructure, and libraries Matplotlib, Numpy, Pandas, Scikit-learn, Scipy, stats-models and VTK for general computation, machine learning and data visualization. Additional code is available at https://github.com/htem/FANC_auto_recon, providing additional tutorials, code and documentation for interacting with FANC.<br><br>https://github.com/sagrawal/Lee_2024<br>https://github.com/htem/FANC_auto_recon

For manuscripts utilizing custom algorithms or software that are central to the research but not yet described in published literature, software must be made available to editors and reviewers. We strongly encourage code deposition in a community repository (e.g. GitHub). See the Nature Portfolio guidelines for submitting code & software for further information.

Data

Policy information about availability of data

All manuscripts must include a data availability statement. This statement should provide the following information, where applicable:

- Accession codes, unique identifiers, or web links for publicly available datasets
- A description of any restrictions on data availability
- For clinical datasets or third party data, please ensure that the statement adheres to our policy

Data presented in the paper was analyzed from the CAVE materialization v604 timestamp 1684915801.222989. Annotated connectivity matrices (Figure 2) will be available as Pandas data frames (https://pandas.pydata.org/) at the GitHub repository: https://github.com/sagrawal/Lee_2024. Links to public segmentations are available throughout the text, as well as in a document at the git-hub repository.

More information can be found at:
https://fanc.community
https://flywire.ai

Field-specific reporting

Please select the one below that is the best fit for your research. If you are not sure, read the appropriate sections before making your selection.

☒ Life sciences ☐ Behavioural & social sciences ☐ Ecological, evolutionary & environmental sciences

For a reference copy of the document with all sections, see nature.com/documents/nr-reporting-summary-flat.pdf

Life sciences study design

All studies must disclose on these points even when the disclosure is negative.

Sample size	Sample size was determined based on data availability and number of neurons we could reconstruct. Neurons in the Female Adult Nerve Cord (FANC) electron microscopy dataset were previously segmented in an automated manner. To manually correct the automated segmentation of our neurons of interest, we used Google's collaborative Neuroglancer interface. Many of the FeCO axons in T1L were previously identified, and most of the claw and hook axons were previously partially corrected. We identified and corrected additional claw axons as well as all club axons in T1L and T2R. Each neuron was categorized as belonging to one of the FeCO subtypes based on their similarity light-level images of FeCO subtype-specific genetic driver lines. An FeCO neuron was deemed "completed" in its reconstruction if all major branches were attached as confirmed by comparison to light microscopy images. All uncertain connections were double-checked by at least two experienced proofreaders, and then a final check of each neuron was completed at the end to again confirm that no false connections were added to a neuron and no major branches were missing.
Data exclusions	We only analyzed pre-synaptic connections of our sensory neurons of interest that made 3 or more synapses onto any given sensory neuron, and postsynaptic connections of our sensory neurons of interest that received 4 or more synapses. Past work found that applying a 3-4 synapse threshold mitigates the inclusion of false positive connections and bias analyses towards stereotyped connections that consistently appear across multiple datasets.
Replication	Data from only an individual fly can be analyzed, because that is the extent of the connectome. We completed analyses to look at how consistent connectivity was across all sensory neurons of a subtype, and also reconstructed the equivalent sensory neurons originating in a different leg and found similar trends
Randomization	The only analysis that utilized randomization was Fig. S2E. Synapses were randomly sampled via custom-written code in python.
Blinding	All analyses were done such that the researcher was blind to the specific sensory neuron. All neurons have an associated number which is the name used by the researcher, which has no clues as to the neuron's type

Reporting for specific materials, systems and methods

We require information from authors about some types of materials, experimental systems and methods used in many studies. Here, indicate whether each material, system or method listed is relevant to your study. If you are not sure if a list item applies to your research, read the appropriate section before selecting a response.

Materials & experimental systems

n/a	Involved in the study
[x]	[ ] Antibodies
[x]	[ ] Eukaryotic cell lines
[x]	[ ] Palaeontology and archaeology
[x]	[ ] Animals and other organisms
[x]	[ ] Human research participants
[x]	[ ] Clinical data
[x]	[ ] Dual use research of concern

Methods

n/a	Involved in the study
[x]	[ ] ChIP-seq
[x]	[ ] Flow cytometry
[x]	[ ] MRI-based neuroimaging
